# Nitrous oxide abuse in a 21-year-old female: a case report and review of literature

**DOI:** 10.3389/fneur.2024.1416557

**Published:** 2024-06-27

**Authors:** Qi Dai, Shutong Chen, Xiaodan Zhang, Kuixin Fan, Jingfeng Zhang, Jianjun Zheng

**Affiliations:** ^1^Department of Radiology, Ningbo No.2 Hospital, Ningbo, China; ^2^School of Medical Imaging, Hangzhou Medical College, Hangzhou, China; ^3^Department of Emergency Medicine, The University of Hong Kong, Hong Kong, China; ^4^Department of Emergency Medicine, Ningbo No.2 Hospital, Ningbo, China

**Keywords:** N_2_O abuse, laughing gas, subacute combined degeneration, vitamin B12, neurological disorders

## Abstract

The abuse of nitrous oxide (N_2_O) poses a substantial public health challenge. In many countries, including China, regulations governing the utilization and accessibility to N_2_O remain ambiguous, particularly within the food industry. Here, we report a case of a 21-year-old female who presented with symptoms of subacute combined degeneration (SCD) of the spinal cord due to N_2_O abuse. The patient exhibited bilateral lower limb numbness and weakness, low serum vitamin B12 levels with elevated homocysteine levels, and lumbar spine magnetic resonance imaging (MRI) revealed abnormal signals of the spinal cord. Following cessation of N_2_O and comprehensive therapy including methylcobalamin and nerve growth factor, the symptoms significantly improved. A follow-up examination 3 months later showed good progress in gait stability. At a 5-year follow-up, the patient’s previous clinical symptoms had completely disappeared, and her quality of life had returned to normal. This case underscores the urgency of raising awareness and prevention of N_2_O abuse, emphasizing the importance of timely diagnosis and comprehensive treatment for patient recovery. Clear formulation and enforcement of relevant regulatory measures are equally crucial in reducing instances of abuse.

## Methods

**Figure 1:**
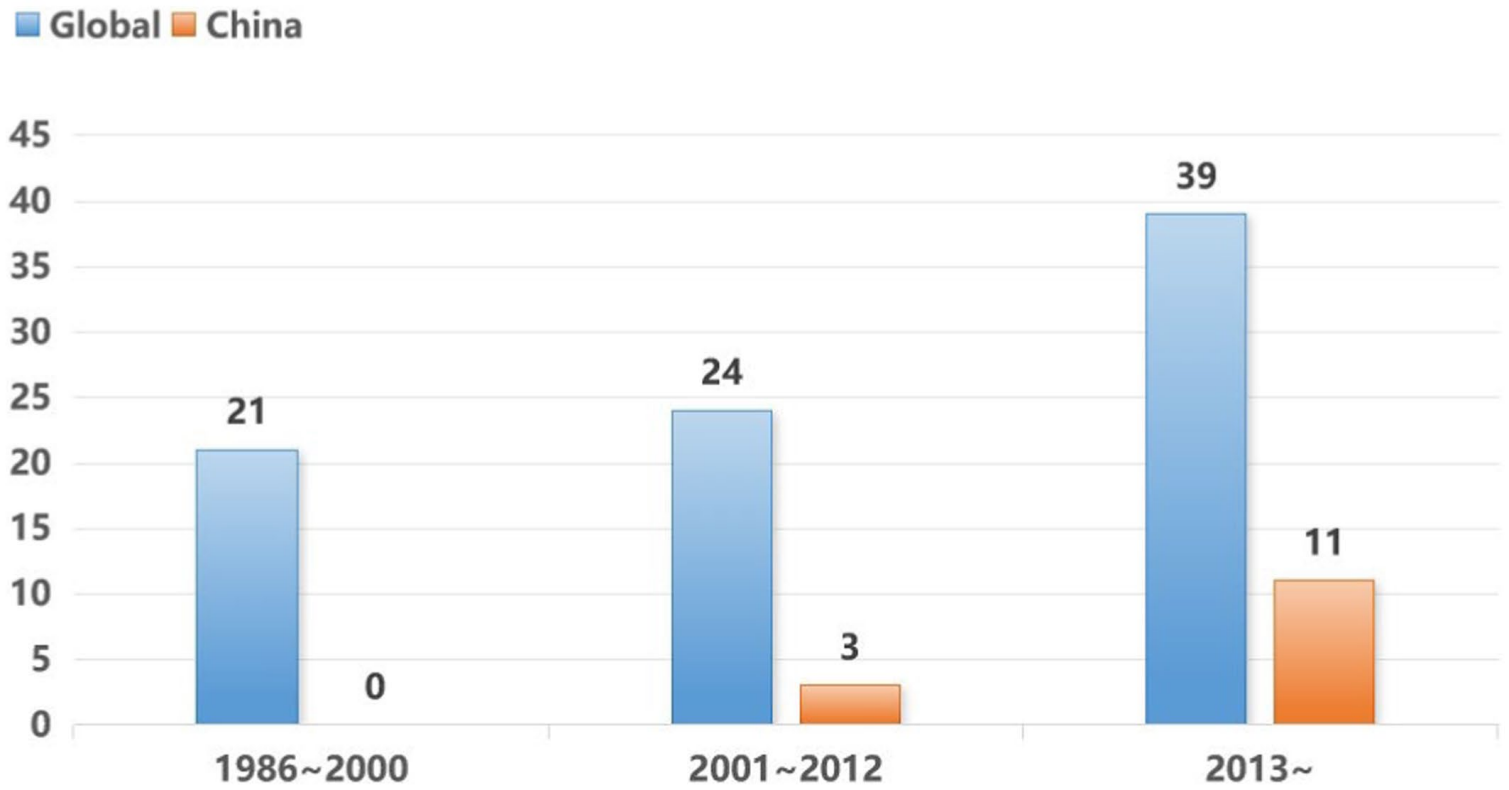
Trends in the number of global and Chinese papers on the topic of N_2_O abuse.

A comprehensive review of the pertinent scientific literature was conducted across databases such as PubMed, Scopus and Web of Science, focusing on English literature. The search strategy employed the terms (“nitrous oxide” OR “laughing gas”) AND (“misuse” OR “abuse” OR “pathogenesis” OR “recreational” OR “neurological diseases”). This rigorous search strategy resulted in a total of 84 publications within the timeframe from January 1986 to April 2024. A discernible upward trend in publication frequency was observed, particularly in the last decade. Notably, 14 papers from Chinese institutions were published post-2001, as illustrated in Figure 1.

## Literature review

In recent years, the abuse of N_2_O has emerged as a significant concern. N_2_O abuse can lead to peripheral neuropathy and central nervous system damage, particularly spinal degenerative disorders, due to vitamin B12 deficiency. According to the 2021 Global Drug Survey (GDS) report, the prevalence of N_2_O use among survey participants reached 22.5%, ranking it thirteenth among recreational drugs (1). N_2_O abuse is notably common among young and middle-aged individuals, with usage rates varying across countries, estimated to range between 8.7 and 12% in 2020 (2, 3). The lifetime incidence of N_2_O abuse-related disorders is approximately 2 to 15.8% (4). The Dutch Drug Information and Monitoring System report an incidence of poisoning at 11% (5). Similar reports have been documented in countries such as China (6, 7), the United States (8), and Italy (9). These findings underscore the global significance of N_2_O abuse, necessitating increased attention and research efforts.

## Clinical presentation

The adverse reactions of N_2_O can manifest as acute neurotoxicity and chronic neurotoxicity. Acute responses include sensory abnormalities (such as numbness, tingling, occurring in 80% of cases), ataxia (58% occurrence), and weakness (43% occurrence) (10). Prolonged use may lead to chronic toxicity, characterized by central nervous system disorders involving the spinal cord and brain (11). Other neurological symptoms may involve urinary and gastrointestinal dysfunctions, memory impairments, headaches, dizziness, and epileptic seizures. Additionally, patients may experience chest tightness (12% occurrence), eating disturbances (9% occurrence), skin allergies (7% occurrence), and sexual dysfunction (5% occurrence) (7). Psychologically, individuals may exhibit anxiety, depression, or psychotic symptoms (12). Reports have indicated that isolated cases may present skin pigmentation starting from the fingers, with extremely rare occurrence of intermittent pigmentation involving the trunk (13).

## Radiologic and laboratory findings

For patients suspected of N_2_O poisoning, routine laboratory and imaging examinations are essential. Laboratory tests include complete blood count, standard biochemical parameters, serum B12 levels, homocysteine, methylmalonic acid, among others. Additionally, standard neurological examinations and autoimmune encephalitis-related antibody tests are common diagnostic methods. Studies indicate that the core manifestation of neurologic damage associated with chronic N_2_O toxicity is disruption in cobalamin metabolism (14). However, at times, serum or active B12 levels may remain normal, suggesting that vitamin B12 deficiency may not be the sole cause of neurological damage (15, 16). Some patients may present with macrocytic anemia, although the correlation between anemia and vitamin B12 deficiency has not been conclusively established (17). Elevated homocysteine levels are a prevalent laboratory abnormality, with around 80% of N_2_O poisoning patients exhibiting elevated homocysteine levels (18). Furthermore, the level of methylmalonic acid is considered as a marker of clinical severity (16).

During imaging examinations, it is essential to include MRI scans of the brain and spinal cord. Research indicates that patients with N_2_O-related Subacute Combined Degeneration (SCD) exhibit more extensive spinal cord lesions on MRI sagittal plane, involving fewer spinal segments, and a higher prevalence of the characteristic “inverted V-sign” on axial MRI. Abnormalities on MRI manifest as high signal intensity in the posterior columns of the spinal cord on T2-weighted imaging, with possible enhancement (19). These features are more pronounced in N_2_O-related SCD patients compared to those without N_2_O exposure (18). Additionally, MRI scans may sometimes show no abnormalities, with studies suggesting a higher likelihood of normal spinal cord MRI in patients exposed to N_2_O for over 6 months (19).

Neurophysiological studies may also provide diagnostic support. An electrophysiological study involving patients with N_2_O-induced peripheral neuropathy, acute inflammatory demyelinating polyneuropathy, acute motor axonal neuropathy, and diabetic peripheral neuropathy revealed significant abnormal neurophysiological features in N_2_O abusers. Electrophysiological examinations could aid in distinguishing N_2_O abuse from other conditions in the diagnostic process (20).

## Diagnostic criteria

Currently, there are no specific international guidelines serving as diagnostic criteria for assessing N_2_O drug abuse and diagnosing subacute combined degeneration of the spinal cord caused by it. Diagnostic approaches currently rely on history taking (including inquiry into long-term or excessive N_2_O use), neurological examination, laboratory and imaging studies, substance abuse assessment, electromyography, and exclusion of other potential causes.

## Case series and case reports

With the widespread increase in recreational use of N_2_O, there has been a growing number of case reports on N_2_O poisoning in recent years. These studies typically enhance understanding and treatment approaches of the condition by analyzing clinical manifestations and outcomes. One study assessed the clinical presentations of 110 patients with N_2_O toxicity, characterized mainly by neurological symptoms such as limb numbness or weakness. Findings revealed that 60% of patients had vitamin B12 deficiency, 69% had elevated homocysteine levels, 92% showed electromyographic evidence of mixed axonal and demyelinating neuropathy, and 24% had spinal cord abnormalities on MRI, aiding in the diagnosis of subacute combined degeneration of the spinal cord. For the majority of patients, N_2_O cessation and vitamin B12 supplementation proved effective in treatment. This study highlights the significance of clinical symptoms and the role of vitamin B12 in therapy (21). Guillaume et al. (22) reported a unique case of severe pancytopenia, polyneuropathy, bilateral pulmonary embolism, and vitamin B12 deficiency associated with hyperhomocysteinemia induced by N_2_O abuse, underscoring the importance of vitamin B12 treatment. Given the complexity of the disease, neurorehabilitation therapy has become increasingly crucial in treatment. Oliver et al. (23) presented a similar case where the patient achieved near-complete recovery with a comparable treatment approach. Another study has documented a case of a patient developing severe motor axonal neuropathy post-N_2_O abuse despite normal levels of vitamin B12, homocysteine, and methylmalonic acid after treatment and recovery from classic vitamin B12 deficiency syndrome. This case suggests that N_2_O-related motor neuropathy may evolve distinctly from vitamin B12 deficiency-related posterior spinal cord disease, adding to the complexity of N_2_O poisoning management (24).

## Treatment

Prolonged abuse of N_2_O without timely treatment can lead to irreversible damage to the nervous system (25). Currently, there are no specific guidelines to direct the management of spinal cord neuropathy induced by N_2_O. Discontinuation of N_2_O use and symptomatic treatment are the primary therapeutic approaches at present. Most patients experience significant symptom relief after several months of N_2_O cessation and supplementation with vitamin B12. Methods of vitamin B12 supplementation include intramuscular hydroxocobalamin injections (1 mg every other day for 2 weeks) or oral supplements of 1,000 μg/day (14, 26, 27). A study has proposed oral naltrexone as a novel treatment strategy to reduce N_2_O cravings, with follow-up observations showing further reduction in N_2_O use after 5 weeks. However, the potential and mechanisms of naltrexone in treating N_2_O addiction require further exploration (28). Some studies support the supplementation of oral L-methionine (1 g three times daily, for at least 4–6 weeks or until significant symptom improvement) as a safe and effective therapy (29, 30). The efficacy of folic acid in N_2_O toxicity treatment remains controversial, with some research suggesting that administering folic acid before vitamin B12 supplementation may worsen symptoms and delay recovery (31, 32). Other studies indicate that supplementing folic acid alongside vitamin B12, but not before, can prevent the deterioration of functional B12 deficiency (29). Combining neurorehabilitation and physical therapy in treatment has shown promising results in optimizing clinical outcomes and promoting patient recovery (22).

## Case report

The patient is a 21-year-old Chinese female with a history of N_2_O misuse. She first experimented with inhaling N_2_O 4 years ago after high school, often referred to as “laughing gas.” She used it sporadically, primarily at social events. She would fill balloons with N_2_O from canisters and inhale them to feel a temporary sense of euphoria. Each use involved approximately 8 grams per canister, with 3–10 canisters per session, happening 2–3 times monthly. In the 6 months leading up to her admission, her N_2_O consumption increased significantly to an average of 1–2 boxes daily, each box containing 300 canisters. The individual’s lifestyle was marked by irregular sleep patterns, subpar dietary habits, and unstable employment. Initially disregarding her health concerns, but upon observing similar behaviors within her social circle, she began to perceive her actions as normal. This highlights the challenges young individuals may face in addressing addictive behaviors and underscores the influence of social factors on individual behavior. It emphasizes the importance of early intervention and psychological support to assist patients in overcoming addiction and improving their quality of life.

In the recent month and a half, she developed symptoms of numbness in her limbs without any apparent trigger. This numbness primarily affected the peripheral regions of her limbs, sometimes accompanied by a tingling sensation. Subsequently, the numbness spread from distal to proximal areas, gradually progressing to numbness in both legs. A week ago, she noticed a decline in coordination while consciously walking, feeling weakness and instability in her lower limbs, leading to multiple falls. During these symptomatic episodes, she did not lose consciousness, nor did she exhibit convulsions or signs of epilepsy. The emergence of these symptoms may link to her prolonged use of N_2_O, underscoring the severity of neurological damage that substance abuse of this nature can entail.

We conducted a comprehensive physical examination and a series of laboratory tests on the patient. The muscle tone of the patient’s limbs exhibited no significant increase or decrease. The proximal strength of both upper limbs was assessed at level 5, while the distal strength was rated at level 4. Both the proximal and distal strength of the lower limbs were rated at level 4 and 4-. Hyperalgesia was noted in both upper and lower limbs below the knees. However, normal vibration and position senses were observed in all limbs. Romberg’s sign was positive regardless of the eyes being open or closed. Tendon reflexes in the upper limbs were within normal limits, but absent in the lower limbs. Hoffmann’s sign was negative bilaterally, and Babinski’s sign was unelicitable. Chaddock’s and Oppenheim’s signs were positive on the right side, but negative on the left. Kernig’s sign yielded a negative result.

The laboratory findings revealed a notable decrease in the patient’s vitamin B12 levels and a significant increase in homocysteine levels. These abnormal results supported the diagnosis of subacute combined degeneration (SCD) of the spinal cord. Other tests including liver and kidney function, complete blood count, immunological markers, and rheumatologic indicators all fell within normal ranges. Table 1 provides a detailed summary of the patient’s key laboratory test results. These findings offer crucial insights into the patient’s condition, aiding in the determination of further diagnostic and treatment strategies.

**Table 1:** The baseline laboratory findings of a 21-year-old female patient prior to treatment initiation.

	Result	Reference interval
**Complete blood count**		
WBC	3.9× 109/L	3.5–9.5 × 109/L
PLT	178 × 109/L	125–350 × 109/L
RBC	3.89 × 1,012/L	3.80–5.10 × 1,012/L
Hb	118 g/L	115–150 g/L
HCT	34.7%	35–45%
MCV	99.5 fL	80–100 fL
RDW	14.9%	11.0–14.5%
**Biochemical parameters**		
B12	107 pg./mL	240–890 pg./mL
Folic Acid	11.5 ng/mL	4.5–10.0 ng/mL
Homocysteine	22.7 μmol/L	4.2–13.5 μmol/L
BUN	3.08 mmol/L	2.60–7.50 mmol/L
Cr	40.5 mmol/L	41.0–73.0 mmol/L
AST	13 IU/L	13–35 IU/L
ALT	12 IU/L	7–40 IU/L
TP	69 g/L	65–85 g/L

WBC, White Blood Cell; PLT, Platelet; RBC, Red Blood Cell; Hb, Hemoglobin; HCT, Hematocrit; MCV, Mean Corpuscular Volume; RDW, Red Cell Distribution Width; B12, Vitamin B12; Folic Acid, Folic Acid; BUN, Blood Urea Nitrogen; Cr, Creatinine; AST, Aspartate Aminotransferase; ALT, Alanine Aminotransferase; TP, Total Protein.

**Figure 2:**
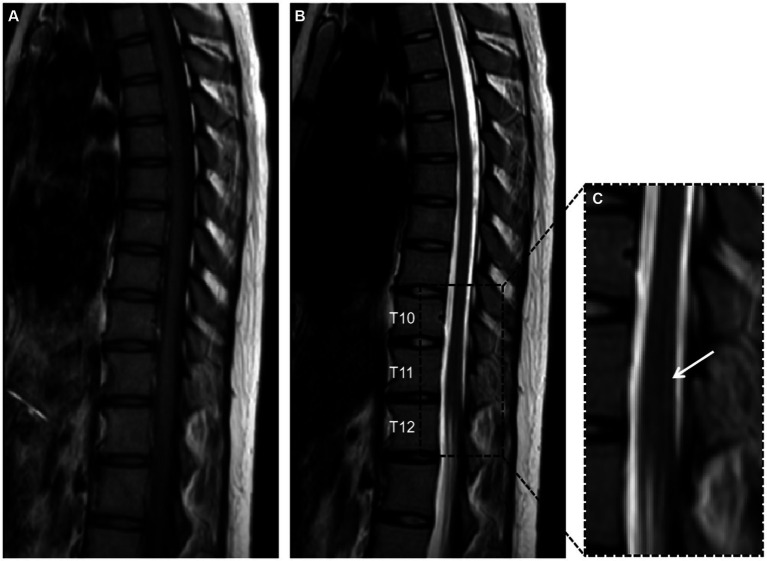
Panels **(A,B)** depict T1-weighted imaging (T1WI) and T2-weighted imaging (T2WI) of the thoracic vertebrae in the sagittal plane, revealing no significant abnormalities. Panel **(C)** provides a detailed magnification of the T10 to T12 segment, showcasing a faint, slender, longitudinally aligned high signal within the posterior compartment of the spinal cord, this phenomenon is recognized as a truncation artifact.

**Figure 3:**
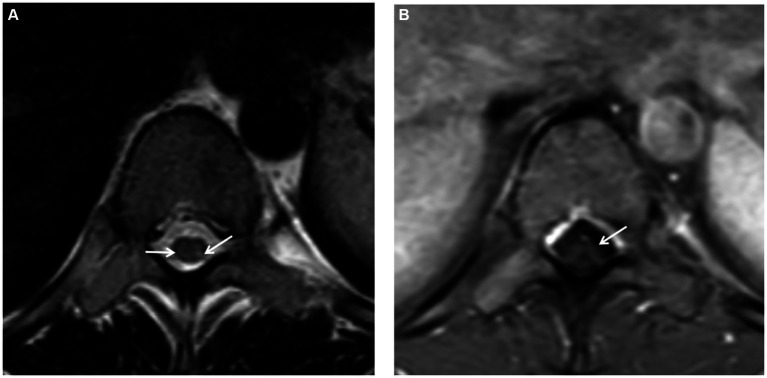
**(A)** On the T2-weighted imaging (T2WI) sequence of the transverse section, small spot-like signals were detected in the posterior and lateral funiculus of the spinal cord at the T10 level, with slightly more significant presence on the left side. **(B)** Following gadolinium contrast enhancement scanning on the T1-weighted imaging (T1WI), a “reverse V-shaped” high signal was evident.

Further spinal MRI was conducted. The T1-weighted imaging (T1WI) sequences (Figure 2A) and sagittal T2-weighted imaging (T2WI) sequences (Figure 2B) demonstrated no significant abnormalities within the thoracic spinal cord. Notably, the subtle, linear, longitudinally oriented hypersignal observed in the posterior spinal area at the T10-T12 level corresponds to a truncation artifact (Figure 2C). Within the T10 horizontal transverse section, T2WI sequence images illustrated small focal abnormal signals localized to the posterior and lateral aspects of the spinal cord (Figure 3A). Subsequent gadolinium-enhanced T1WI scanning at this level revealed a prominent “inverted V” pattern with high signal intensity (Figure 3B). This distinctive imaging characteristic, coupled with the patient’s signs of neurological impairment, corroborates the diagnosis of subacute combined degeneration of the spinal cord. Furthermore, comprehensive imaging evaluations, encompassing brain and cervical MRI, chest CT scans, and cardiac and abdominal ultrasounds, did not uncover any additional abnormalities.

Based on the patient’s history of N_2_O abuse and various examination indicators, the initial clinical diagnosis revolved around toxic myelopathy, particularly subacute combined degeneration (SCD) of the spinal cord. Drawing from research on the pathophysiology of N_2_O toxicity, we developed a comprehensive treatment plan tailored to the patient’s condition. Following cessation of N_2_O inhalation, the patient underwent treatment including daily intravenous injections of 500 μg methylcobalamin, daily intramuscular injections of 18 μg nerve growth factor, daily oral intake of 0.50 mg clonazepam, intramuscular injections of 1,000 mg hydroxocobalamin every other day, and daily oral administration of 5 mg folic acid after discontinuing B12. Additionally, we implemented a nutritional neurosupportive regimen, incorporating supplements such as oral amino acids to facilitate the repair of degenerating myelin sheaths, along with a structured functional exercise program spanning 4 weeks. These integrated therapeutic interventions aim to comprehensively improve the patient’s symptoms and functional recovery, enhancing the effectiveness of treatment.

Subsequent assessments revealed a significant improvement in the patient’s limb muscle strength and a reduction in numbness, ultimately leading to the patient’s discharge. During the following three-month follow-up period, despite initially experiencing gait instability, the patient continued to make notable progress. The observed enhancements in motor function and sensory symptoms further substantiated the effectiveness of the implemented treatment strategies in addressing the neurotoxic sequelae induced by N_2_O. A follow-up conducted 5 years later showed the complete disappearance of the patient’s previous symptoms, with a return to a normal quality of life.

## Discussion

N_2_O is a colorless gas commonly utilized in interventional therapy due to its analgesic and sedative effects. Popularly known as “laughing gas,” inhalation of N_2_O induces feelings of euphoria, with rapid onset and short duration of action. It can also enhance the bubbles and flavors of beverages, making it increasingly popular as a recreational substance among young individuals. While the legitimate purchase of N_2_O for medical and culinary purposes is permitted, there is a growing trend of illegal recreational use worldwide (33). In Western countries, up to 29.4 to 38.6% of individuals in the United Kingdom and the United States will use N_2_O in their lifetime. The demographic primarily engaging in recreational N_2_O use consists of adolescents, with statistics indicating an average age of around 24.3 years among abusers (34), with over 80,000 young people misusing N_2_O annually. Generally, the amount of N_2_O inhaled correlates with the severity of neurological symptoms. However, some patients manifest significant neurological symptoms despite inhaling only small amount of N_2_O. In clinical practice, when the amount of N_2_O inhaled during surgery is below 40 grams, the risk is relatively low. Nonetheless, exceeding 80 grams per day significantly increases the risk of permanent nerve damage, such as an exponential rise in the risk of ataxic paraplegia (35). Epidemiological studies suggest that N_2_O may be a risk factor for infertility, miscarriage, fetal malformations, and tumors. The abuse of N_2_O can lead to toxicity, ultimately resulting in irreversible nerve damage. Therefore, the abuse of N_2_O must be given due attention to mitigate its potential harm to individual health.

Upon inhalation, N_2_O is swiftly absorbed through the pulmonary circulation. As a molecule with high lipid solubility, it easily traverses the blood–brain barrier and primarily exerts its effects via the opiate system by directly binding to mu, delta, and kappa opiate receptors (1). Additionally, N_2_O may function as an NMDA receptor antagonist similar to ketamine (2). A single inhalation of N_2_O typically does not induce neurotoxic alterations unless pre-existing vitamin B12 levels are significantly deficient. Its action is characterized by a rapid onset and it is eliminated from the body within hours. The fact that it is not detectable by most standard drug testing kits likely contributes to its widespread misuse.

**Figure 4:**
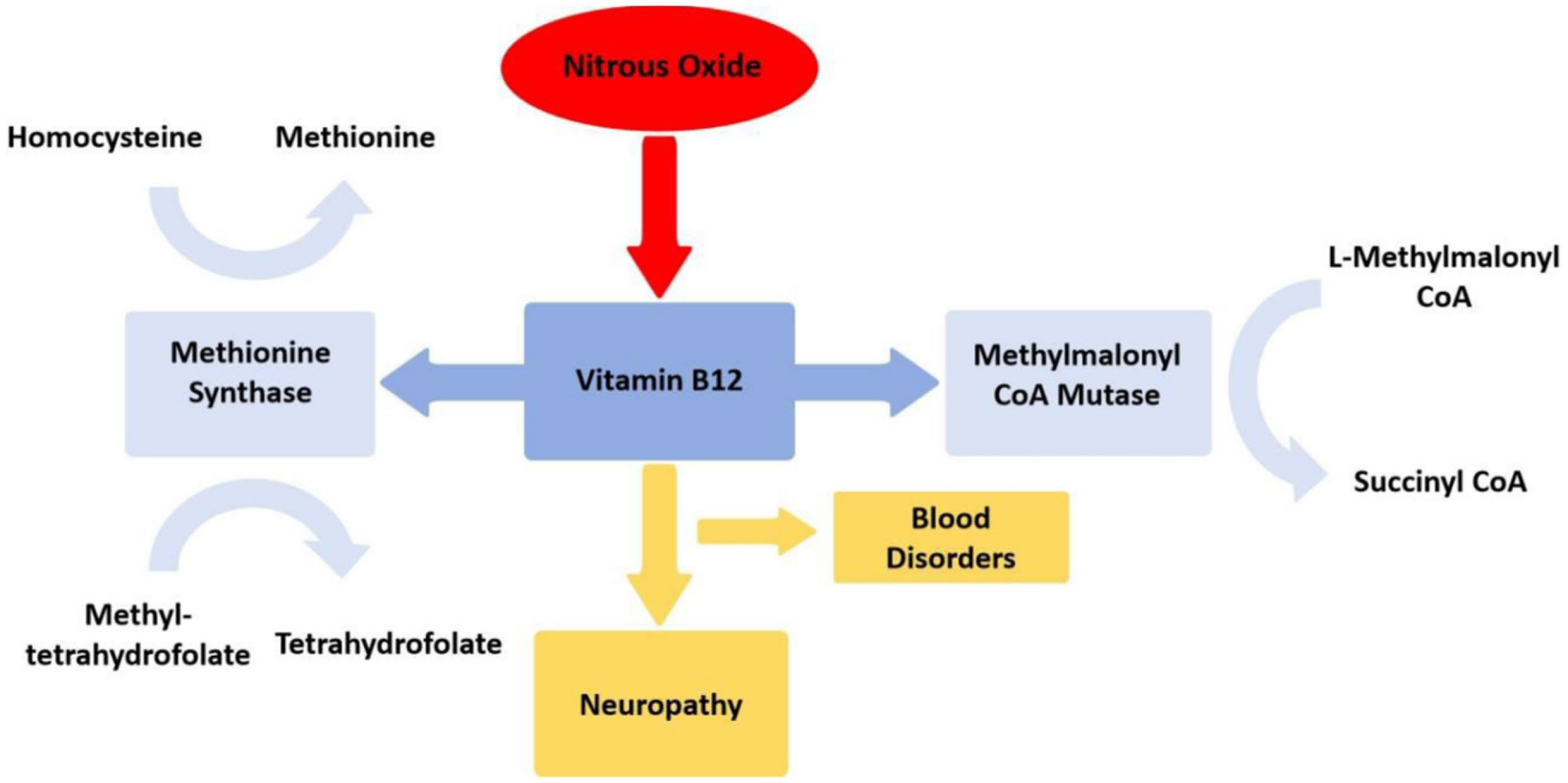
Mechanism of nitrous oxide disrupting vitamin B12 metabolism. Mecobalamin serves as a crucial coenzyme for methionine synthase, facilitating the conversion of homocysteine to methionine and 5-methyltetrahydrofolate to tetrahydrofolate. Additionally, adenosylcobalamin functions as an indispensable cofactor for methylmalonyl-CoA mutase, which catalyzes the transformation of methylmalonyl-CoA into succinyl-CoA.

The neurological and psychiatric symptoms induced by N_2_O are primarily associated with its impact on the body’s vitamin B12 levels (4). Vitamin B12 exists in the body in the form of methylcobalamin and serves as a cofactor for the enzymes methionine synthase and methylmalonyl-CoA mutase, participating in the metabolism of blood and the nervous system (36). Methionine synthase converts homocysteine into methionine, an intermediate product for DNA synthesis, essential for the production and metabolism of nerve myelin sheaths; methyl tetrahydrofolate is converted into tetrahydrofolate, further transforming into physiologically active methylcobalamin, contributing to blood cell formation and neurotransmitter synthesis; while methylmalonyl-CoA mutase converts L-methylmalonyl-CoA into succinyl-CoA (33). N_2_O oxidizes the cobalt ion of vitamin B12 to a + 2 oxidation state irreversibly, leading to the inactivation of methylcobalamin, disrupting the aforementioned metabolic reactions, impairing DNA and myelin sheath synthesis, resulting in blood disorders (e.g. megaloblastic anemia) and neurological defects (4, 33) (Figure 4). Nearly all neurological symptoms induced by N_2_O are accompanied by vitamin B12 deficiency (12, 30, 37), further supporting the influence of N_2_O on vitamin B12. Without timely supplementation of vitamin B12, excessive use of N_2_O can lead to the accumulation of homocysteine and methylmalonic acid, causing subacute combined degeneration of the spinal cord (38). These hypotheses align with reports of motor disturbances in patients. Studies suggest that individuals abusing N_2_O exhibit pronounced motor hyperexcitability changes and less apparent sensory hyperexcitability changes, indicating that N_2_O may cause axonal functional impairments through its toxic effects on the extramedullary region (39), offering new insights for disease diagnosis and treatment.

Through the integration of medical history, clinical manifestations, laboratory examinations, and imaging studies, our patient was ultimately diagnosed with subacute combined degeneration of the spinal cord caused by the abuse of N_2_O. The predominant symptoms manifested as limb numbness and ataxia, typical of the pathology induced by vitamin B12 deficiency. Laboratory tests play a crucial role in diagnosing N_2_O toxicity. As previously mentioned, nearly all N_2_O-induced neurological symptoms are associated with signs of vitamin B12 deficiency, with most patients exhibiting slightly elevated levels of homocysteine. This further confirms that N_2_O primarily disrupts cellular cobalamin metabolism, while cobalamin levels in peripheral blood may remain within normal limits in the early stages of the disease (40). Imaging findings vary depending on the affected areas. Some case reports indicate that N_2_O toxicity predominantly affects the corticospinal tracts and posterior columns of the spinal cord in the lower cervical and upper thoracic segments (41). On T2-weighted MRI, there is a high signal intensity with a “reversed V-shaped” along the axis. Enhanced imaging typically does not reveal any enhancement patterns. In this case, the MRI abnormalities were predominantly observed in the thoracic submedullary segment. The T2 sequence revealed slight irregularities, while the T1-enhanced sequence images exhibited characteristic abnormalities within a limited involvement range, suggesting a favorable clinical prognosis.

Currently, there are no definitive international guidelines providing direction on the mechanisms of neurotoxicity caused by N_2_O and its treatment. Commonly employed basic therapies include supplementation with vitamin B12 and folic acid. Our treatment approach involves discontinuation of N_2_O exposure, high-dose vitamin B12 supplementation, and initiation of oral supplements such as cystine 4 weeks after cessation to promote myelin sheath repair. Most symptoms show improvement within the initial 6 months (42). The response to treatment depends on factors such as the severity of the condition, timing of treatment initiation, younger age, milder nerve damage observed on spinal cord MRI, absence of anemia, and the likelihood of symptom recovery (43). Based on previous case reports, the majority of patients can regain normal physiological function or experience only minor residual neurological deficits following aggressive treatment. However, some individuals may suffer severe and irreversible nerve damage due to continued exposure or irregular treatment, leading to loss of independence in daily living, and in extreme cases, cardiac arrest and sudden death (44). In this case, supplementation with vitamin B12 and symptomatic treatment improved bilateral muscle tone abnormalities, graphomotor skills, and significantly enhanced proximal muscle strength, while instability in sitting and standing was noted; pathological signs were negative. Complete recovery of distal muscle strength has not yet been achieved, likely representing short-term sequelae that may gradually improve over time with ongoing rehabilitation exercises.

The identification of neurodegeneration induced by N_2_O must be differentiated from other diseases. In tests of motor and sensory nerve excitability, these patients exhibit more pronounced changes in motor excitability and weaker changes in sensory excitability compared to individuals with isolated vitamin B12 deficiency (39). When compared to pure spinal degenerative disease, the neurodegenerative effects caused by N_2_O toxicity primarily stem from the direct toxic effects of N_2_O on the nervous system, resulting in damage to blood and nerve systems that may reach irreversible levels. Conversely, pure neurodegenerative disease is a chronic, progressive nervous system disorder typically associated with genetic, environmental, and lifestyle factors. Therefore, in the realm of treatment, addressing the toxic symptoms promptly and emphasizing the supplementation of vitamin B12 are crucial for promoting the recovery of neural function in cases of neurodegeneration induced by N_2_O poisoning.

In the international medical and entertainment spheres, the abuse of N_2_O has emerged as a significant global public health concern. Noteworthy is the fact that abusers of N_2_O extend beyond the general population, as historical cases have documented instances of excessive use among certain professionals (33). To prevent the misuse or abuse of N_2_O, it is imperative to garner the attention of medical professionals and intensify educational efforts. Clinical practice often focuses more on N_2_O’s adverse reactions as an inhaled anesthetic, neglecting the serious harm it can cause as a recreational substance. Particularly, anesthesiologists and surgeons should pay heed to the severity of recreational N_2_O exposure in the general population (10), while also judiciously regulating the dosage and frequency of medical N_2_O use. For patients requiring N_2_O anesthesia, a preoperative assessment of their physical condition and tolerance is recommended. Generally, N_2_O anesthesia is not affected by the synthesis of homocysteine, provided that patients have sufficient reserves of vitamin B12 (45). For high-risk groups (such as the elderly, pregnant women, individuals with hypertension, and diabetes patients), proactive supplementation of vitamin B12 should be considered before surgery to reduce the risk of postoperative hyperhomocysteinemia (46). In cases where N_2_O poisoning is suspected, a systematic clinical evaluation should be conducted, encompassing physical examinations (neurological assessments), laboratory tests (complete blood count, standard biochemical markers, serum B12 levels, homocysteine, methylmalonic acid, routine cerebrospinal fluid analysis, and testing for antibodies related to autoimmune encephalitis), and imaging studies (brain and spinal MRI).

Over the past few years, uncommon accidents and fatalities associated with nitrous oxide misuse on university campuses and among young individuals have been extensively reported by the media, thereby increasing political awareness in multiple countries. Consequently, as of 8 November 2023, it has become illegal in the United Kingdom to possess, supply, import, export or produce nitrous oxide outside of its intended purposes (47). The long-term impact of this regulatory change, along with similar legal measures in other regions, on the prevalence of N_2_O abuse, associated harm, toxicity, and related deaths remains to be observed. In addition to the efforts of healthcare institutions, there is an urgent need to enhance the awareness and vigilance of social groups such as schools and nursing personnel to minimize adolescent exposure to N_2_O. Strict control measures must be imposed on the production and distribution of N_2_O by relevant authorities to curb its abuse. In the face of the daunting reality of N_2_O abuse, concerted efforts from global community are still required.

## Author contributions

QD: Conceptualization, Investigation, Methodology, Writing – original draft, Writing – review & editing. SC: Formal analysis, Writing – original draft. XZ: Investigation, Writing – review & editing. KF: Supervision, Writing – review & editing. JZha: Conceptualization, Supervision, Writing – review & editing. JZhe: Formal analysis, Funding acquisition, Investigation, Writing – review & editing.
